# Targeting upstream alarmins (TSLP, IL-33, IL-25): a new frontier in treating type 2 chronic rhinosinusitis with nasal polyps

**DOI:** 10.3389/fimmu.2026.1970480

**Published:** 2026-10-09

**Authors:** Shuhua Li

**Affiliations:** Department of Otolaryngology, Mianyang Central Hospital/The Affiliated Mianyang Hospital of University of Electronic Science and Technology, Mianyang, Sichuan, China

**Keywords:** alarmins, biologics, chronic rhinosinusitis with nasal polyps, IL-25, IL-33, precision medicine, TSLP, type 2 inflammation

## Abstract

Chronic rhinosinusitis with nasal polyps (CRSwNP) affects 1%-4% of adults globally and imposes a substantial burden on quality of life. Despite endoscopic surgery, topical corticosteroids, and systemic glucocorticoids, postoperative recurrence exceeds 54% at five years. Even with the advent of biologics targeting downstream type 2 effectors, such as dupilumab, mepolizumab, and omalizumab, a meaningful subset of patients, particularly those with mixed or non-type 2 endotypes, remains inadequately controlled. The epithelial alarmins thymic stromal lymphopoietin (TSLP), interleukin-33 (IL-33), and interleukin-25 (IL-25) occupy the apex of the inflammatory cascade, functioning as proximal triggers that bridge innate barrier disruption to adaptive type 2 amplification. This review examines the shared and distinctive pathobiology of these three molecules in CRSwNP, compares the clinical trajectories of tezepelumab (Phase 3), anti-IL-33/ST2 agents (Phase 2/3), and emerging anti-IL-25 biologics (early-phase), and outlines a conceptual framework for future investigation. We propose that upstream alarmin inhibition offers a broader immunomodulatory scope than downstream cytokine blockade, yet its clinical translation hinges on three unresolved challenges: overcoming functional redundancy among alarmins, defining the long-term safety profile of sustained epithelial-immune suppression, and validating clinically accessible predictive biomarkers. The proposed algorithms and biomarker cut-offs remain exploratory and require prospective validation in CRSwNP-specific cohorts.

## 1 Introduction

CRSwNP is not a homogeneous disorder but rather a family of inflammatory endotypes unified by chronic mucosal eosinophilia in most Western populations. The socioeconomic burden is substantial, driven by lost productivity, repeated clinic visits, and surgical interventions (1). In European and North American cohorts, type 2 inflammation, orchestrated by CD4+ T helper 2 (Th2) cells, group 2 innate lymphoid cells (ILC2s), and eosinophils, accounts for approximately 80% of cases. A similar trend is now emerging across Asia (2, 3). The effector cytokines IL-4, IL-5, and IL-13 sustain a self-perpetuating cycle of eosinophil recruitment, mucus hypersecretion, and submucosal fibrosis (4). Yet even with maximal medical therapy and functional endoscopic sinus surgery, roughly half of patients experience polyp regrowth within five years, a statistic that has shown little improvement over the past decade (5, 6).

The introduction of biologics that target downstream effectors has broadened the therapeutic landscape. Dupilumab (anti-IL-4 receptor α), mepolizumab (anti-IL-5), and omalizumab (anti-immunoglobulin E) have each demonstrated efficacy in randomized controlled trials. Nevertheless, response rates are neither universal nor complete. A substantial proportion of patients, especially those with low eosinophil counts or overlapping neutrophilic components, derive only marginal benefit (7). This clinical ceiling has prompted a strategic refocusing on the earliest events in the type 2 inflammatory cascade, namely the release of epithelial-derived alarmins (TSLP, IL-33, and IL-25) upon barrier injury, allergen exposure, or microbial challenge.

These molecules form the critical interface between innate immune sensing and adaptive type 2 polarization. They engage TSLPR, ST2 (also known as IL-1 receptor-like 1), and the IL-17RA/IL-17RB heterodimer on dendritic cells, ILC2s, and Th2 cells, respectively (8, 9). Because they occupy the apex of the inflammatory hierarchy, their concurrent inhibition could theoretically suppress both T2-high and T2-low pathways simultaneously. This breadth of action sets upstream inhibition apart from single-cytokine neutralization, yet the clinical development of these approaches has progressed unevenly: tezepelumab is the only alarmin-targeted biologic with positive Phase 3 data in CRSwNP, whereas anti-IL-33/ST2 agents remain in Phase 2/3 trials and anti-IL-25 candidates are still in early-phase exploration.

In the nasal epithelium of CRSwNP, genetic predisposition and environmental stressors converge to drive overexpression of all three alarmins (10, 11). Compared with downstream cytokine blockade, targeting these upstream molecules offers the prospect of broader immunomodulation and potentially more durable disease control (12–14). Yet this promise is tempered by substantial challenges. Functional redundancy among the three alarmins may undermine the efficacy of monotherapy. Systemic neutralisation of epithelial-derived signals raises legitimate concerns about impaired tissue surveillance and infection risk. Furthermore, robust and clinically accessible biomarkers to guide patient selection remain conspicuously absent.

In this review, we synthesize the current preclinical and clinical evidence on alarmin-directed therapies in CRSwNP, critically examine the translational bottlenecks that have impeded progress, and delineate a hypothesis-generating framework that could inform future trial design and biomarker discovery. This narrative review was based on searches of PubMed and ClinicalTrials.gov up to mid-2026. Search terms included combinations of ‘alarmins,’ ‘TSLP,’ ‘IL-33,’ ‘IL-25,’ ‘CRSwNP,’ and ‘biologics.’ Studies were selected based on relevance to the mechanistic or clinical aspects of alarmin-targeted therapy in CRSwNP. No systematic screening or quality assessment was performed, consistent with the narrative format.”

## 2 Alarmin biology in type 2 CRSwNP: convergence and divergence

TSLP, IL-33, and IL-25 share a common purpose: to alert the immune system to epithelial compromise. Yet they differ fundamentally in their triggers, receptor usage, cellular targets, and temporal dynamics. Far from being a purely intellectual exercise, delineating these differences carries direct implications for biologic prioritization.

### 2.1 Thymic stromal lymphopoietin

TSLP is a four-helix-bundle cytokine belonging to the IL-2 family. It signals through a heterodimeric receptor composed of TSLPR and IL-7 receptor α (IL-7Rα) (15, 16). Its expression is governed by nuclear factor kappa-B (NF-κB) and various epithelial stressors. Within nasal polyp tissue, TSLP transcript levels correlate strongly with eosinophil counts and composite measures of disease severity (17, 18).

Functionally, TSLP operates primarily through dendritic cells. It licenses these antigen-presenting cells to prime naive CD4+ T cells toward a Th2 phenotype, while also exerting direct activating effects on ILC2s (19). The subsequent secretion of IL-4, IL-5, and IL-13 orchestrates eosinophilic infiltration, goblet cell metaplasia, and stromal remodelling—the triad of pathological changes that defines CRSwNP (20, 21). A clinically relevant nuance is that proteolytically truncated TSLP variants retain full biological activity yet frequently escape detection by standard immunoassays. This measurement bias complicates efforts to establish reliable biomarker cut-offs (22). Of the three alarmin targets, TSLP is the most clinically advanced, with the anti-TSLP antibody tezepelumab having completed a positive Phase 3 trial in CRSwNP (discussed in Section 2).

### 2.2 Interleukin-33

IL-33 occupies a unique position among the alarmins. Under steady-state conditions, it resides constitutively in the nuclei of epithelial and endothelial cells, where it participates in transcriptional regulation. Upon cellular necrosis or stress-induced secretion, it transitions to an extracellular alarmin, signalling through ST2 (23, 24). This dual functionality, whereby IL-33 acts as a nuclear regulator in homeostatic conditions and functions as a proinflammatory alarmin following cellular release, distinguishes it from both TSLP and IL-25, which lack such dual biological properties.

The functional consequences of this compartmentalized biology are twofold.IL-33/ST2 engagement potently activates ILC2s and Th2 cells to produce IL-5 and IL-13, thereby promoting eosinophilia and mucus metaplasia. Beyond this, IL-33 also directly stimulates fibroblast proliferation and extracellular matrix deposition, thereby coupling acute type 2 inflammation to chronic fibrotic remodelling in nasal polyps (25). This pro-fibrotic effect appears more directly attributable to IL-33 than to TSLP or IL-25, neither of which has been shown to exert comparable direct actions on stromal cells in CRSwNP tissue. Clinically, IL-33’s nuclear-to-extracellular shuttling may explain the weak correlation between circulating IL-33 and tissue inflammation, limiting the value of plasma IL-33 as a standalone biomarker. Anti-IL-33 and anti-ST2 agents are currently in Phase 2/3 development, with results more heterogeneous than those reported for tezepelumab.

### 2.3 Interleukin-25

IL-25, also designated IL-17E, signals through a heterodimer of IL-17RA and IL-17RB. IL-17RB expression is enriched on ILC2s, Th2 cells, and activated B cells within polyp tissue (26). The cellular provenance of IL-25 differs markedly from that of TSLP and IL-33; it is predominantly produced by epithelial tuft cells and mast cells rather than by epithelial cells broadly (27).

The major biological effect of IL-25 is goblet cell hyperplasia and mucus hypersecretion, pathways that directly contribute to nasal obstruction and polyp formation (27). IL-25 has also been implicated in corticosteroid-refractory disease in some experimental models, raising the possibility—though still unconfirmed in CRSwNP—that patients with steroid-insensitive phenotypes might derive benefit from IL-25 blockade, should such agents become available (28). Unlike TSLP and IL-33, anti-IL-25 antibodies have not yet advanced to registrational trials in CRSwNP, and their clinical development remains at an early stage.

### 2.4 Critical crosstalk and functional redundancy

Importantly, these alarmins are released in parallel rather than in a sequential linear cascade, and their effects on target cells are partially overlapping. Co-expression studies in nasal polyp tissue reveal that TSLP, IL-33, and IL-25 synergize to amplify ILC2-derived cytokine output, creating a positive feed-forward loop that perpetuates tissue damage (9, 29, 30). Yet the relative dominance of each alarmin varies considerably across individuals. Some patients show predominant TSLP upregulation, whereas others show elevated IL-33 or IL-25 signalling. This variation is shaped by genetic background (e.g., ST2 polymorphisms), local microbial ecology, and comorbid conditions such as asthma or aspirin-exacerbated respiratory disease (AERD) (31–33).

The signalling reach of TSLP and IL-33 extends beyond classic type 2 circuitry. TSLP can activate myeloid dendritic cells to produce tumour necrosis factor-α (TNF-α) and interleukin-6 (IL-6), while IL-33 potentiates mast cell-derived pro-inflammatory mediators independently of ILC2s (34, 35). This expanded repertoire may explain why tezepelumab retains efficacy in low-eosinophil phenotypes, a feature that distinguishes upstream alarmin blockade from downstream cytokine neutralisation. The interactions among TSLP, IL-33, and IL-25 and their downstream signaling in the nasal epithelial barrier are summarized (**Figure 1**). One unresolved question is whether blocking a single alarmin triggers compensatory upregulation of the remaining two; if so, monotherapy might have limited efficacy. We return to this possibility in Section 5, where we consider sequential and combination strategies as hypothetical approaches.

**Figure 1:**
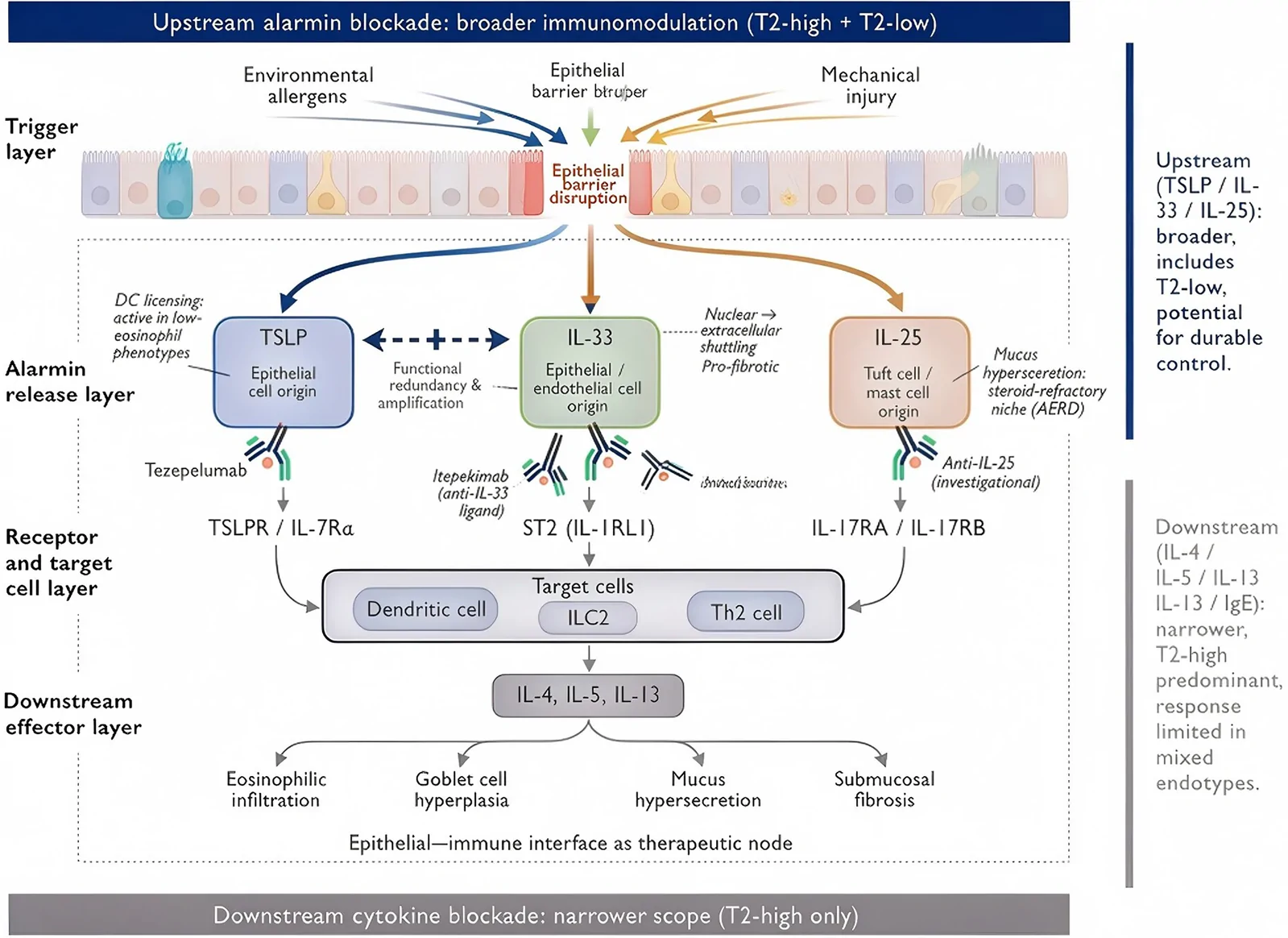
Epithelial alarmin pathways in type 2 CRSwNP: parallel release, functional redundancy, and upstream versus downstream therapeutic targeting.

## 3 TSLP-targeted therapies: from asthma success to CRSwNP validation

### 3.1 Tezepelumab: pivotal trial evidence and real-world data

Tezepelumab is a fully human immunoglobulin G2 (IgG2) monoclonal antibody that binds TSLP with high affinity, preventing its interaction with the TSLPR/IL-7Rα complex (36). It remains the first and currently the only alarmin-targeted biologic licensed for the treatment of severe asthma (37). By intercepting TSLP, it attenuates both dendritic cell- and ILC2-driven pathways, reducing downstream IL-4, IL-5, and IL-13 production irrespective of baseline eosinophil counts (38).

The NAVIGATOR trial provided the foundational evidence in asthma, demonstrating significant exacerbation reduction across a broad range of phenotypes. *Post-hoc* analyses of the comorbid CRSwNP subgroup showed favourable sinonasal outcomes, including improvements in the 22-item Sino-Nasal Outcome Test (SNOT-22) and polyp scores (38). These signals paved the way for a dedicated CRSwNP trial.

The WAYPOINT study, a Phase 3 randomized controlled trial published in *NEJM* in 2025, enrolled 408 adults with severe uncontrolled CRSwNP. Participants were assigned to tezepelumab 210 mg or placebo every four weeks for 52 weeks. At week 52, the tezepelumab arm achieved a mean reduction in total nasal polyp score of –2.08 (95% confidence interval [CI], –2.40 to –1.76) and a nasal congestion improvement of –1.04 (95% CI, –1.21 to –0.87; both P<0.001) (38). All secondary endpoints, including olfaction, SNOT-22, and Lund–Mackay computed tomography scores, improved significantly. These improvements were accompanied by marked reductions in rescue surgery and systemic glucocorticoid bursts (38). These data unequivocally establish tezepelumab as an effective biologic for severe CRSwNP, particularly in patients who have previously failed downstream-targeted therapies.

Real-world case series have since corroborated these findings, documenting concurrent improvement in asthma control and sinonasal symptoms among highly comorbid patients (39). Mechanistically, tezepelumab’s reach may extend beyond pure type 2 immunity. Emerging evidence suggests activity in low-T2 inflammatory states, which could explain its consistent performance across diverse patient subsets—a feature that may extend efficacy to patients with baseline eosinophil counts below the thresholds typically required for dupilumab or mepolizumab (36).

### 3.2 Unresolved questions and biomarker development

While the WAYPOINT trial data definitively demonstrate tezepelumab’s therapeutic efficacy, several pivotal uncertainties remain unresolved, which will shape its optimal clinical positioning. First, the proposed plasma TSLP threshold of 330 fg/mL, derived from the DUBHE trial, remains an exploratory cut-off that has not been prospectively validated in CRSwNP (40). It is crucial to emphasize that this threshold is an exploratory cut-off that has not been prospectively validated in CRSwNP, and it should not be used as a clinical decision-making tool outside of research settings. Whether this threshold identifies true responders or merely reflects baseline disease severity is unclear. Second, although short-term safety appears favourable, with no signal of increased serious infections, long-term immunogenicity and the theoretical risk of impaired epithelial tumour surveillance require ongoing pharmacovigilance. Third, whether TSLP blockade outperforms alternative mechanisms in pure T2-high versus mixed endotypes is unknown; answering this question will require dedicated head-to-head comparisons.

### 3.3 Alternative modalities and delivery approaches

Small-molecule inhibitors and antisense oligonucleotides targeting TSLP messenger RNA (mRNA) are in preclinical stages (41). Topical nasal spray formulations are conceptually attractive for minimizing systemic exposure, but they face substantial technical hurdles in mucosal penetration and formulation stability (42). In our view, until these oral or topical alternatives demonstrate non-inferiority in adequately powered trials, systemic tezepelumab will remain the clinical backbone.

## 4 Targeting the IL-33/ST2 axis: progress, pitfalls, and paradoxes

### 4.1 Ligand-directed strategies: etokimab and itepekimab

In contrast to tezepelumab, anti-IL-33/ST2 agents have not yet demonstrated consistent efficacy in Phase 2/3 trials, and no agent in this class has positive Phase 3 data in CRSwNP. Etokimab (ANB020), a first-generation anti-IL-33 antibody, generated early enthusiasm in atopic dermatitis and asthma, where it reduced type 2 inflammatory markers (38, 43). In Phase 2 CRSwNP studies, however, its effects on polyp size and nasal symptoms were inconsistent. A subset of patients showed minimal or no response, while others derived meaningful benefit (44). This heterogeneity likely reflects the predominance of IL-33-independent pathways in certain endotypes, underscoring the critical need for pre-treatment biomarkers, such as tissue ST2 expression or soluble ST2 (sST2) levels, to enrich responsive populations.

Itepekimab (REGN3500), a more mature anti-IL-33 candidate, is currently under Phase 3 evaluation in CRSwNP. In asthmatic patients, it significantly reduced exacerbations and improved lung function (45, 46). However, a combination trial pairing itepekimab with dupilumab failed to consistently demonstrate additive benefit over dupilumab alone across all endpoints. This could mean that IL-33 and IL-4Rα pathways converge on shared downstream nodes, or that the trial was underpowered for subgroup analyses (47). Combination targeted therapy may still offer clinical benefits for specific patient subgroups, particularly those with refractory airway disease and robust baseline ST2/IL-33 inflammatory signatures. Nevertheless, this mechanistic hypothesis requires prospective clinical validation alongside qualified companion diagnostic tools.

### 4.2 Receptor-directed strategy: astegolimab

Rather than ligand neutralisation, therapeutic targeting of the receptor represents an alternative approach, as illustrated by astegolimab. Astegolimab (MSTT1041A) takes a fundamentally different approach by blocking the ST2 receptor rather than the IL-33 ligand. This strategy offers several theoretical advantages. It neutralizes both IL-33 isoforms and receptor-amplified signals, and it does not interfere with the nuclear functions of IL-33 (48). Early-phase studies in chronic obstructive pulmonary disease (COPD) and asthma have shown acceptable safety and preliminary efficacy signals (49).

In CRSwNP, ST2 blockade could prove more robust than ligand neutralisation, particularly given the existence of truncated IL-33 variants with enhanced ST2 affinity. Conversely, ST2 is also expressed on regulatory T cells (Tregs), and its blockade could perturb Treg homeostasis, a trade-off that warrants close monitoring in ongoing trials (50). Whether this receptor-directed approach will yield superior clinical outcomes to ligand neutralisation remains an open question. Adding to this complexity, the IL-33 signalling axis is regulated by the soluble decoy receptor sST2, which may confound biomarker analyses and influence therapeutic responsiveness (51).

### 4.3 The soluble ST2 paradox and its clinical implications

sST2 acts as a decoy receptor to sequester free IL-33, inhibiting IL-33/ST2L signalling (52). Paradoxically, increased circulating sST2 correlates with disease severity in asthma and cardiovascular disorders, yet its standalone predictive value for CRSwNP biologic response remains ambiguous. In CRSwNP patients, serum sST2 levels are significantly elevated, particularly in eosinophilic endotypes, and are associated with tissue eosinophil infiltration and postoperative recurrence (53). High circulating sST2 may reflect two mutually exclusive clinical scenarios: exaggerated IL-33-driven mucosal inflammation with compensatory decoy receptor production, or effective systemic sequestration of bioactive IL-33—scenarios that carry opposite therapeutic implications for anti-IL-33/ST2 treatment. To resolve this ambiguity, we hypothesise that the free IL-33/sST2 molar ratio may reflect IL-33/ST2 axis activation and therapeutic response more accurately than absolute sST2 levels (54). Because sST2 is a high-affinity decoy receptor, total IL-33 measurements misrepresent bioavailable ligand, and elevated sST2 may reflect either compensatory decoy production or effective neutralisation—each carrying opposite implications for anti-IL-33 therapy (52). This ratio has not been prospectively validated in any CRSwNP cohort and remains a theoretical proposal. Technical challenges include the lack of standardized assays for free versus total IL-33 and the absence of clinically accessible measurement platforms. This hypothesis is testable in ongoing trials and warrants prospective exploration as an exploratory biomarker.

Notably, tissue-based mRNA profiling via nasal polyp biopsy or minimally invasive nasal brushing yields higher predictive accuracy than peripheral blood biomarkers, as local alarmin concentrations evade systemic dilution artefacts, and nasal brushing transcriptomics has been successfully applied to identify molecular endotypes in CRSwNP and to predict therapeutic response (55). Nevertheless, tissue sampling faces real-world clinical barriers: polyp biopsy carries minor bleeding risks, while nasal brush sampling lacks standardized operating protocols across clinical centres, limiting large-scale routine implementation. Peripheral plasma TSLP and free IL-33/sST2 ratios represent practical non-invasive alternatives for preliminary patient screening. A two-step diagnostic workflow is therefore proposed: peripheral blood biomarker screening for preliminary stratification, followed by nasal brush molecular profiling to confirm dominant alarmin endotypes before biologic initiation. This tiered strategy balances diagnostic precision and clinical accessibility for routine rhinology practice, though it awaits prospective validation.

## 5 IL-25 blockade: a developmental latecomer with distinct niche potential

### 5.1 Current clinical landscape

Of the three alarmin targets, anti-IL-25 antibodies have lagged furthest behind in clinical development. In asthma, the IL-17RA-blocking antibody brodalumab (which disrupts IL-25 signaling) failed a phase 2 trial and did not improve asthma control or lung function (56). In CRSwNP, IL-25 is overexpressed in polyp tissue and has been proposed as a biomarker of airway hyperreactivity, but no anti-IL-25 biologic has advanced beyond early-stage exploration (57). Several IL-25-directed agents show preclinical activity, yet none have translated into consistent clinical benefit in type 2 airway diseases. Mechanistically, IL-25 contributes to eosinophilic inflammation and is induced by HIF-1α in the hypoxic polyp microenvironment; blocking IL-25 attenuates these pathways in ECRSwNP models (58). The translational gap probably reflects several factors, including unvalidated biomarkers, uncertain endpoints, and the possibility that IL-25 acts mainly in disease initiation rather than maintenance, though these remain hypotheses. We return to these issues in Section 4.3.

### 5.2 Receptor-targeting combinations: promise and peril

Given that IL-25 shares the IL-17RA subunit with IL-17A and IL-17F, combined IL-25/IL-17A blockade presents an intriguing concept for mixed type 2/type 17 endotypes (59). However, IL-17RA is also a critical mediator of mucosal antifungal immunity, and its blockade carries a consistent risk of mucocutaneous candidiasis and other extracellular infections, as observed with anti-IL-17A therapies in psoriasis (60). We recommend that such dual strategies be reserved for carefully phenotyped patients with documented mixed neutrophilic/eosinophilic polyps, and only under strict infection surveillance protocols.

### 5.3 Explanations for the developmental lag

Three factors likely account for IL-25’s slower progress. First, its functional overlap with TSLP and IL-33 may permit pathway escape when IL-25 alone is blocked. Second, IL-25 may be more instrumental in initiating rather than sustaining established polyp disease, a temporal distinction that current trial designs have not systematically addressed. Third, the tuft-cell origin of IL-25 renders its production more episodic and luminal-dependent, unlike the constitutive epithelial release of TSLP and IL-33 (61). This characteristic makes single time-point measurements less reliable. These challenges do not invalidate IL-25 as a therapeutic target, but they call for more dynamic sampling approaches and refined endotyping strategies.

Aspirin-exacerbated respiratory disease (AERD) represents an instructive case in point. In addition, AERD is also a highly refractory CRSwNP subtype. It exhibits constitutively expanded tuft cell populations and exaggerated IL-25 secretion independent of eosinophil burden (9, 61). This unique molecular phenotype partially accounts for the pronounced mucus hyperplasia and frequent steroid refractoriness observed in AERD patients. In AERD, IL-25 is released from epithelial-associated tuft cells via a cysteinyl leukotriene-dependent pathway and is elevated in patient plasma, whereas TSLP and IL-33 are also upregulated but may operate through partially distinct mast cell- and lipid-mediated mechanisms. These observations suggest that IL-25 could be a particularly relevant driver in this subset; however, whether IL-25 blockade confers unique clinical benefits remains a hypothesis awaiting prospective validation in dedicated trials. Current trial designs have insufficiently stratified AERD versus non-AERD cohorts, which further obscures the true clinical potential of IL-25-targeted therapies in real-world refractory CRSwNP.

## 6 Towards a precision framework: a hypothesis-generating model for future research

The following framework is conceptual and derives from an integration of mechanistic insights and limited clinical data. It has not been validated in prospective interventional studies and should not be interpreted as a clinical practice guideline.

### 6.1 Comparative landscape and molecular endotype framework

Three alarmin-targeted approaches are currently being evaluated clinically for CRSwNP: anti-TSLP (tezepelumab), anti-IL-33/ST2 (etokimab, itepekimab, astegolimab), and anti-IL-25. Each strategy possesses a distinct biological rationale, and these differences in clinical development stage and evidence strength are summarized (**Supplementary Figure 1**), with detailed mechanistic comparisons provided in **Table 1**.

**Table 1:** Comparative landscape of alarmin-targeted agents in CRSwNP: mechanisms, evidence levels, and biomarker candidates.

Feature	TSLP (Tezepelumab)	IL-33 (Itepekimab)	IL-33 (Astegolimab)	IL-25
Target	Anti-TSLP ligand	Anti-IL-33 ligand	Anti-ST2 receptor	Anti-IL-25 ligand
Primary cellular source	Epithelial cells	Epithelial / endothelial cells (constitutive nuclear expression)	Epithelial / endothelial cells (constitutive nuclear expression)	Tuft cells, mast cells
Receptor complex	TSLPR / IL-7Rα	ST2 (IL-1RL1)	ST2 (IL-1RL1)	IL-17RA / IL-17RB
Dominant downstream effect	DC → Th2 priming; ILC2 activation	ILC2 / Th2 → IL-5/IL-13; fibroblast activation → fibrosis	ILC2 / Th2 → IL-5/IL-13; fibroblast activation → fibrosis	ILC2 → IL-5/IL-13; goblet cell hyperplasia
Clinical stage in CRSwNP	Phase 3 positive (WAYPOINT) ✅	Phase 3 ongoing ⚠	Phase 2b/3 ongoing ⚠	Phase 1/2 exploration ❌
Evidence status	Approved in asthma; positive pivotal data in CRSwNP	Mixed Phase 2 results; no positive pivotal data to date	Mixed Phase 2 results; no positive pivotal data to date	No positive registrational efficacy data
Key biomarker candidate	Baseline plasma TSLP (330 fg/mL, exploratory)	Free IL-33 / sST2 ratio (hypothetical)	Free IL-33 / sST2 ratio (hypothetical)	Tissue IL-17RB expression; Tuft cell markers
Distinct advantage	Broadest efficacy across eosinophil strata; robust RCT data	Targets both inflammation and fibrotic remodelling	Targets both inflammation and fibrotic remodelling	May address corticosteroid-insensitive mucus hypersecretion
Distinct limitation	Limited data on non-T2 efficacy in CRSwNP	Variable trial results; combination with dupilumab underwhelming	Variable trial results; combination with dupilumab underwhelming	No positive pivotal data; high redundancy risk

Ongoing or exploratory; no positive pivotal data to date. Itepekimab targets the IL-33 ligand; astegolimab targets the ST2 receptor. Both belong to the same axis but differ in mechanism. None of the biomarker candidates listed has been prospectively validated in CRSwNP cohorts. Evidence status reflects the strongest available data as of mid-2026.

Evidence status key: ✅ positive pivotal data; ⚠ mixed/inconclusive results; ❌ no positive data. All biomarker candidates are exploratory and require prospective validation in CRSwNP-specific cohorts.

Tezepelumab, backed by the positive WAYPOINT trial, currently enjoys the strongest clinical evidence currently available, with efficacy that extends across eosinophil strata. In the phase 3 WAYPOINT trial, tezepelumab reduced total nasal polyp score by –2.07 (95% CI, –2.39 to –1.74) and nasal congestion score by –1.03 (95% CI, –1.20 to –0.86) compared with placebo at week 52, with significant improvements in SNOT-22 (–27.26), Lund–Mackay score (–5.72), and loss-of-smell score (–1.00) (62). Surgery for nasal polyps was indicated in only 0.5% of tezepelumab-treated patients versus 22.1% of placebo recipients (HR 0.02), and systemic glucocorticoid use was reduced from 18.3% to 5.2% (HR 0.12) (62). A network meta-analysis confirmed tezepelumab’s robust efficacy across multiple sinonasal outcomes, with the greatest improvement in SNOT-22 among all evaluated biologics (63). This feature distinguishes it from downstream inhibitors. Anti-IL-33 agents are mechanistically appealing due to their dual anti-inflammatory and anti-fibrotic potential, but inconsistent trial outcomes and underwhelming dupilumab combination data temper enthusiasm. Anti-IL-25 remains the least advanced; while no pivotal positive data exist, its possible role in corticosteroid-resistant mucus hypersecretion defines a unique niche worth investigating (64).

The rationale for targeting alarmins rather than downstream cytokines extends beyond simple pathway interception. TSLP, for instance, can activate myeloid dendritic cells to produce TNF-α and IL-6 independently of classical Th2 signalling, which may explain its efficacy in patients with low eosinophil counts. TSLP and TSLPR expression levels are significantly elevated in both peripheral blood and nasal polyp tissues of CRSwNP patients, supporting their potential utility as minimally invasive biomarkers (17). Notably, TSLP has been linked to other inflammatory conditions beyond type 2 immunity, including non-eosinophilic endotypes, which may account for its broader efficacy profile (36). This broader immunological reach is mechanistically distinct from IL-4/IL-13 or IL-5 blockade, and it forms the basis for considering alarmin inhibitors as a separate therapeutic class rather than merely an alternative to existing biologics.

Biomarker development must prioritize actionable classification over statistical association. However, it is critical to recognize that no alarmin-based biomarker has yet been prospectively validated as a predictor of treatment response in CRSwNP. Against this background, we outline a hypothesis-generating stratification model that could inform future trial design rather than current clinical practice. We propose stratifying CRSwNP into two broad categories based on tissue alarmin mRNA profiles from nasal brushings or polyp biopsies. Tissue-based measurements are likely to outperform plasma levels because local concentrations are higher and systemic dilution is minimized, though both approaches remain investigational.

The first category includes alarmin-dominant endotypes, which can be further subdivided into TSLP-high, IL-33-high, and IL-25-high according to the predominant mRNA signature. Within this proposed model, tezepelumab would be the most biologically rational candidate, pending prospective validation, pending prospective confirmation. For an IL-33-high signature, an anti-IL-33 or anti-ST2 agent such as itepekimab or astegolimab would be the mechanistically aligned candidate. For an IL-25-high signature, anti-IL-25 strategies may be considered where available in clinical trials (64), though the absence of pivotal efficacy data makes this the most speculative arm of the framework.

The second category includes non-alarmin-dominant endotypes, in which none of the three alarmins is markedly upregulated. This group encompasses mixed or non-T2 phenotypes, and alarmin inhibitors may be less likely to provide benefit, although this hypothesis requires prospective testing. Instead, these individuals could be considered for alternative immunomodulators or receive management of modifiable comorbidities including obesity, smoking, and uncontrolled asthma. This suggestion remains speculative and requires prospective evaluation. If validated, such an algorithm could improve the number needed to treat by directing biologics toward patients most likely to respond, a consideration with potential health-economic implications.

The framework should not be applied rigidly. Comorbid asthma, AERD status, sputum eosinophil counts, and serum periostin levels should all be integrated into a composite decision algorithm rather than being used as standalone binary predictors (65). The plasma TSLP threshold of 330 fg/mL derived from the DUBHE trial represents a starting point for further investigation rather than a validated clinical cut-off. Prospective validation in CRSwNP-specific cohorts is urgently needed.

The comparative landscape outlined above and summarized in **Table 1** provides the biological and clinical rationale for target selection, but it does not by itself offer a practical roadmap for the practising clinician. To translate these mechanistic distinctions into actionable treatment decisions, we propose a two-tier biomarker-driven algorithm (**Figure 2**). This framework integrates peripheral blood screening, tissue-based molecular confirmation, and early response assessment to guide first-line biologic selection and escalation decisions.

**Figure 2:**
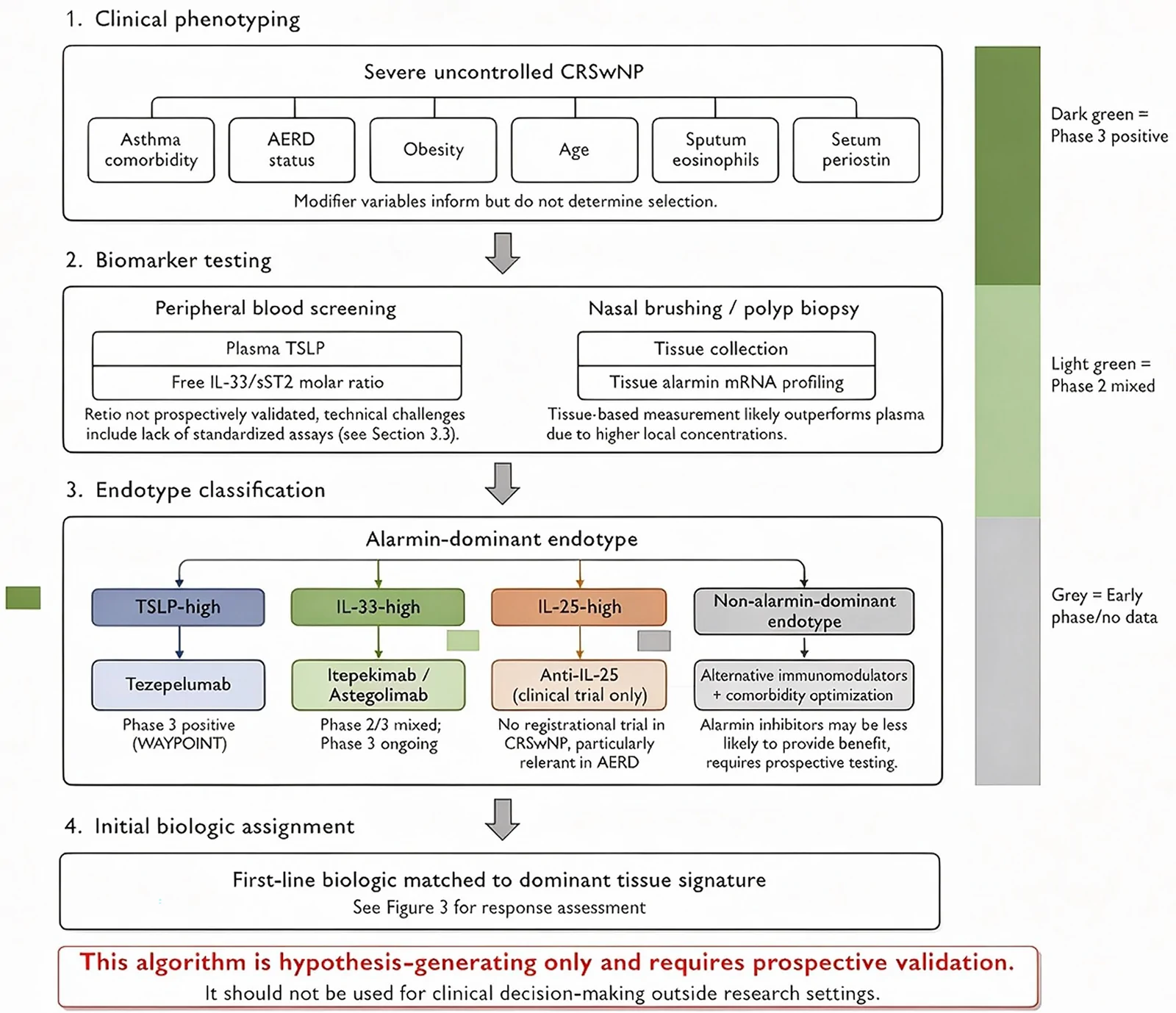
Proposed conceptual framework for endotype-guided first-line alarmin-targeted biologic selection in severe uncontrolled CRSwNP. Not validated in prospective studies.

### 6.2 Functional redundancy and the case for sequential combination

The sequential stratification model described below is a conceptual proposal and has not been tested in clinical trials. A key unresolved question, as noted in Section 1.4, is whether blocking a single alarmin leads to compensatory upregulation of the others. Preclinical data from asthma models provide some support for this concern, as IL-33 blockade led to relative TSLP elevation in certain tissues (8). Novel bispecific antibody platforms represent an emerging modality to overcome alarmin functional redundancy, with two core designs advancing into preclinical and early-phase clinical development: anti-TSLP/IL-13 bispecific IgG1 (CM512) and dual TSLP/IL-4Rα neutralizing constructs (66, 67). These molecules intercept both upstream alarmin priming and downstream effector signalling in a single construct, theoretically breaking compensatory inflammatory feedback loops without requiring two separate infusions.

Despite their mechanistic appeal, dual alarmin co-blockade carries theoretical concerns regarding sustained epithelial–immune modulation, which could compromise mucosal anti-pathogen surveillance and tumour immune monitoring. These concerns are theoretical and have not been demonstrated in clinical trials; they justify long-term safety surveillance rather than being viewed as established risks. As an alternative to upfront combination, we propose a “sequential stratification” model as a conceptual approach. Patients could be initiated on a single alarmin inhibitor chosen according to the dominant tissue expression signature. Those who fail to achieve an early response—for example, persistent polyp oedema or insufficient improvement in patient-reported outcomes—could then be considered for escalation to dual blockade in a trial setting. We propose, as a hypothesis for future testing, that response assessment at 16–24 weeks using a SNOT-22 improvement threshold of ≥8 points could serve as an exploratory decision point. Neither the timing nor the threshold has been validated, and both should be evaluated prospectively in CRSwNP-specific cohorts. This adaptive design, though currently untested, might offer advantages in safety and cost-effectiveness by reserving dual blockade for the minority who truly require multi-target suppression. This sequential stratification model is summarized in **Figure 3**.

**Figure 3:**
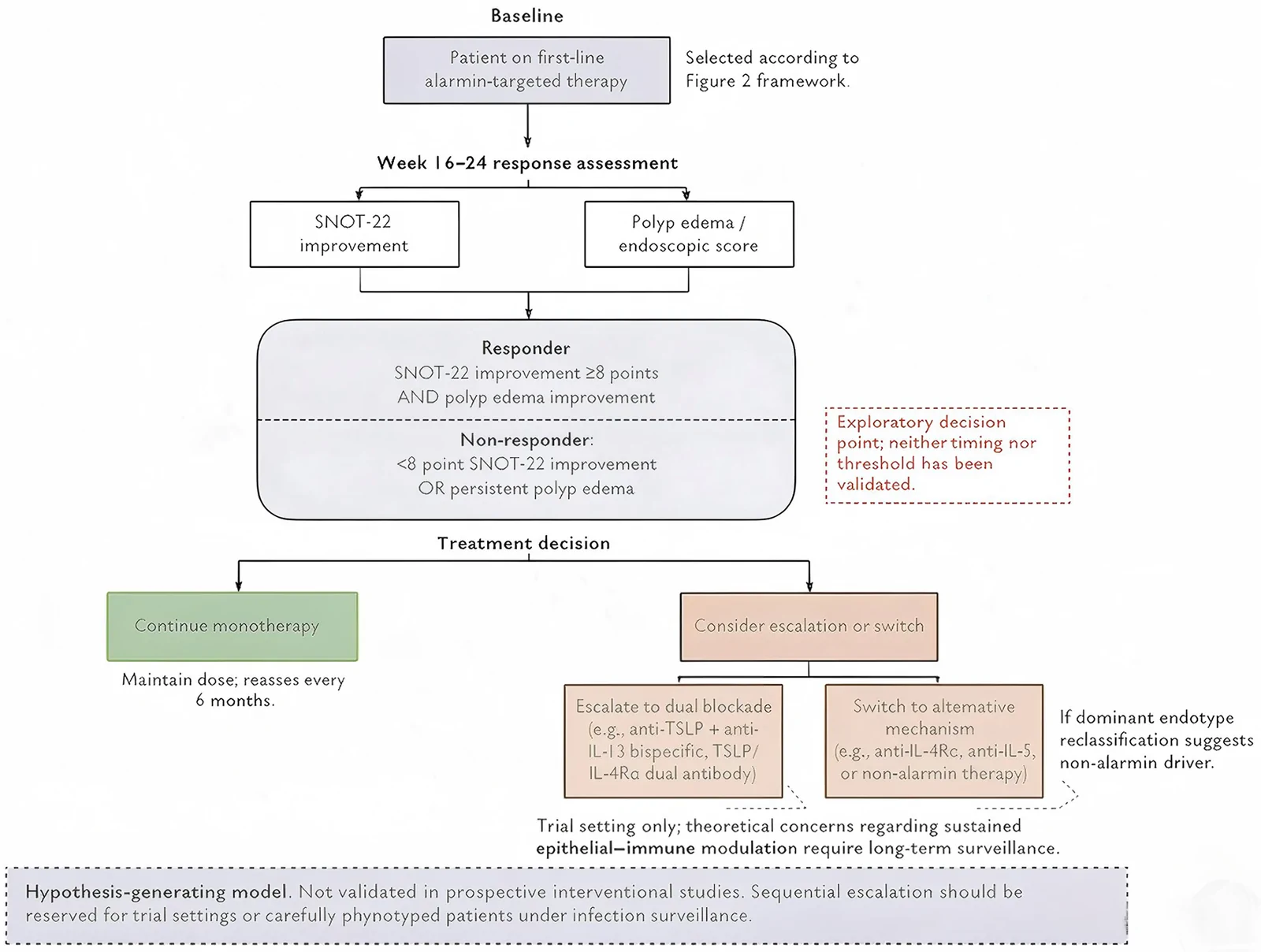
Proposed conceptual framework for response assessment and sequential escalation after first-line alarmin-targeted therapy in CRSwNP. Hypothesis-generating; not validated.

### 6.3 Long-term safety and delivery innovation

Current trial durations, which extend at most to 52 weeks, are insufficient to assess rare adverse events, immunogenicity, and theoretical risks that justify long-term surveillance, including the possibility of malignant transformation associated with chronic epithelial–immune suppression. Real-world registries, such as the EPOS2020 biologics registry, should extend follow-up to five years or more to capture these signals. Paediatric and elderly populations are critically underrepresented in existing trials—a gap that warrants attention given that asthma and atopic diatheses often manifest in early childhood, and early alarmin intervention could theoretically alter the natural history of disease (68).

Several host factors may modify treatment response. Obese patients with CRSwNP tend to have lower systemic levels of IL-33 and TSLP, suggesting a predominantly type 2-low endotype (32). These patients may respond differently to alarmin blockade, although this hypothesis requires dedicated study and should not be used to exclude obese patients from biologic therapy.

On the delivery front, nanoparticle-encapsulated formulations and sustained-release injectables have shown promise in murine allergic models but face substantial translational hurdles, including mucosal barrier penetration, endosomal entrapment, and batch-to-batch consistency (69). Until these challenges are resolved, emphasis should remain on optimizing dose intervals and loading strategies for existing antibodies.

## 7 Conclusion

Tezepelumab’s Phase 3 success supports the viability of upstream alarmin interception in CRSwNP, alongside existing downstream biologics. The interconnected biology of TSLP, IL-33, and IL-25 offers a versatile therapeutic arsenal, yet their functional overlap demands rigorous biomarker-driven patient selection and, in selected cases, sequential regimens. Critical challenges remain, including long-term safety surveillance, validation of tissue-based predictive signatures, and integration of host factors such as obesity, microbiome, and genetics into clinical algorithms.

We propose that future strategies should move toward adaptive precision medicine, in which target selection, response monitoring, and treatment escalation are guided by molecular phenotyping. Should this framework be validated in prospective cohorts, it could yield durable disease modification in severe recurrent CRSwNP. The translational concepts outlined here, including tissue-based endotyping, the free IL-33/sST2 ratio as a biomarker model, and sequential treatment escalation, require prospective validation across diverse populations, including Western, Asian, paediatric, and elderly cohorts.
